# Seed Priming with 2,4-Epibrassionolide Enhances Seed Germination and Heat Tolerance in Rice by Regulating the Antioxidant System and Plant Hormone Signaling Pathways

**DOI:** 10.3390/antiox14020242

**Published:** 2025-02-19

**Authors:** Jingya Qian, Xu Mo, Yue Wang, Qiang Li

**Affiliations:** 1 College of Agronomy, Hunan Agricultural University, Changsha 410128, China; qianjingya@stu.hunau.edu.cn; 2 The Key Laboratory of Crop Germplasm Innovation and Resource Utilization of Hunan Province, Hunan Agricultural University, Changsha 410128, China; mx525@stu.hunau.edu.cn; 3 Yuelu Mountain Laboratory of Hunan Province, Hunan Agricultural University, Changsha 410128, China

**Keywords:** rice seeds, 2,4-epibrassionolide, seed initiator, heat stress, antioxidant system, plant hormone signaling

## Abstract

With global climate warming, enhancing the heat stress tolerance of rice seeds is critical for ensuring crop yields and maintaining global food security. 2,4-Epibrassionolide (EBR) has been shown to effectively alleviate the adverse effects of heat stress on rice seed germination, but its mitigation mechanism has not been fully clarified. In this experiment, exogenous EBR was used as a seed priming agent. The activities of superoxide dismutase (SOD), peroxidase (POD), catalase (CAT), malondialdehyde (MDA), soluble protein contents, and plant hormone levels were measured during rice seed germination under heat stress (38 °C). We constructed a cDNA library for transcriptome sequencing analysis. The results showed that exogenous EBR could effectively alleviate the effect of heat stress on rice seeds by enhancing SOD, POD, and CAT enzyme activity; reducing the MDA content; and increasing the soluble protein content. Additionally, exogenous EBR increases the levels of GA and IAA while decreasing the ABA content. According to a transcriptomic analysis, exogenous EBR can induce the expression of key genes involved in GA, IAA, and ABA hormone biosynthesis and metabolism, regulating GA-, IAA-, ABA-, and H_2_O_2_-mediated signaling pathways to promote the germination of rice seeds under heat stress. This study provides new insights into the application of rice seed priming techniques.

## 1. Introduction

Due to the increasing population and the acceleration of industrialization, a large number of greenhouse gases, such as carbon dioxide (CO_2_) and methane (CH_4_), are being emitted into the atmosphere, and the global temperature is gradually increasing [1] (p. 151). The continuous increase in surface temperature threatens future global food security, and this threat will become even more serious as the population grows. Therefore, improving the heat stress tolerance in food crops to ensure safe production is an urgent issue.

Heat stress can profoundly impede seed germination by disrupting a range of physiological processes essential for seed growth and development. The activity of antioxidant enzymes is particularly critical during germination, as enzymes such as amylases, proteases, and lipases facilitate the hydrolysis of stored nutrients into bioavailable forms necessary for embryo growth [2,3]. Under heat stress conditions, the enzymatic activity of these proteins is often diminished, thereby slowing the metabolic processes vital for successful seedling development. Furthermore, the integrity of cellular membranes is a key determinant of seed viability, and exposure to elevated temperatures can provoke oxidative stress, leading to the accumulation of reactive oxygen species (ROS). These ROS inflict cellular damage that compromises the functionality of cellular structures and impedes the initiation of germination. Moreover, previous studies have demonstrated that heat stress can disrupt the hormonal equilibrium within seeds, particularly by enhancing the synthesis of abscisic acid (ABA), a plant hormone known to inhibit germination under stress conditions. This disruption in hormonal balance can result in delayed or suppressed seed germination [4].

Seed priming is an important method in agricultural production that is used to solve the problem of slow and uneven seedling emergence. This technology was first introduced by Heydecker in 1973. It involves soaking seeds under controlled conditions to allow them to absorb water and swell, followed by re-drying (back-drying). During this process, seed germination is regulated by controlling the moisture content of the seeds. It is a common pre-sowing seed treatment technique in agricultural production [5]. The key to seed initiation is to allow the seed to fully absorb water, break dormancy, activate and fully complete a series of metabolic activities required for germination, and reach the state of imminent germination [6,7]. It can activate the physiological and metabolic activities related to germination in seeds, promoting the repair of cell membranes, organelles, and DNA. This provides more favorable conditions for seed germination, particularly improving resistance to adversity stress during germination and seedling emergence. This technique has been shown to be effective in improving the vigor of crop seeds. Tomato [8], watermelon [9], and maize [10] seeds have been primed to significantly enhance germination and adversity tolerance.

Brassinosteroids (BRs) are sterols commonly found in plants. They have high-efficiency, broad-spectrum, and non-toxic physiological activities and are recognized as the sixth class of plant hormones. 2,4-Epibrassionolide (EBR) is one of the most active compounds among the brassinosteroids. It plays a key role in regulating plant growth and development and alleviating stresses caused by various biotic and abiotic factors [11]. In one study, when 1 µmol/L EBR was added to a medium, plant growth was significantly restored with hypocotyl elongation, petiole growth, and obvious increases in leaf area [12]. EBR can regulate photosynthesis light reactions and carbohydrate metabolism both negatively and positively by increasing enzyme activities in the Calvin cycle [13]. Exogenous EBR has been shown to improve leaf area, specific leaf area, stem–leaf pinch angle, stem–leaf droop angle, and stem–leaf droop in tomatoes; it also increases apparent quantum efficiency, the dark respiration rate, carboxylation efficiency, Rubisco content, and effectively mitigates damage from low-light stress in tomato seedlings [14]. In Arabidopsis and *Brassica napus*, it induces the expression of a series of key genes under drought and cold stress that can enhance tolerance to drought and cold stress [15]. Notably, in our previous experiments, we found that using exogenous EBR as a seed priming agent had a significant effect in improving the tolerance of rice seeds to heat stress. It was most effective at a concentration of 0.05 µmol/L [16]. However, the physiological and molecular mechanisms of its heat stress alleviation are uncertain. Therefore, we took XWX13 as the test material and used a 0.05 µmol/L EBR solution as the seed primer. We further investigated the effects of exogenous EBR on antioxidant enzymes, malondialdehyde, soluble proteins, and phytohormones in rice seeds under heat stress, as well as physiological mechanisms. The main objectives of our study are as follows: (1) to analyze the effects of exogenous EBR on the physiological characteristics and phytohormone content of rice seeds; (2) to reveal the mechanism of exogenous EBR in alleviating heat stress in rice seeds; (3) and to provide technical references for improving exogenous EBR and promoting its application in rice seed production as a seed priming agent.

## 2. Materials and Methods

### 2.1. Plant Material

The material examined was XWX13, a late-maturing rice cultivar primarily cultivated in rice-producing regions along the middle and lower reaches of the Yangtze River in China.

### 2.2. Experimental Design

Plump rice seeds of the same size were selected, sterilized with 0.5% sodium hypochlorite for 20 min, and then rinsed three times with distilled water. After seed sterilization, the seeds were soaked in EBR solution at a concentration of 0.05 µmol/L and incubated at 25 °C for 24 h in a light incubator (RLD-1000E-4, Ningbo Ledian Instrument Manufacturing Co., Ltd., Ningbo, China). We used sterilized distilled water priming as the control (CK). At the end of the seed priming, the seeds were washed with sterilized distilled water and blotted dry with sterilized filter paper. We dried the seeds back to the initial moisture content by placing them in a blast drying oven (DHG-9140A, Shanghai Yiheng Scientific Instruments Co., Ltd., Shanghai, China) at 25 °C. After priming, the seeds were uniformly sown in germination cassettes (12 cm × 12 cm × 5 cm in length, width, and height, respectively) lined with sterilized germination paper. Each germination box was sown with 100 seeds. We conducted the seed germination test in a light incubator. The heat stress treatment was set at 38 °C, 16 h light–8 h dark, and 70% humidity. The seeds were germinated based on shoot length equal to half the seed length and root length equal to the seed length. We measured germination potential and germination percentage on days 3 and 7 of germination. The germination potential was determined using the following formula: Germination potential (%) = (number of seeds germinated on day 3/total number of experimental seeds) × 100. Similarly, the germination rate was calculated as follows: Germination rate (%) = (number of seeds germinated on day 7/total number of experimental seeds) × 100. Following 24 h of heat stress treatment, seed embryos pretreated with sterile distilled water (HT) and 0.05 µmol/L EBR solution (HT+EBR) were collected to evaluate for antioxidant enzyme activity, malondialdehyde (MDA) content, soluble protein concentration, plant hormone levels, transcriptomic profiling, and the expression of relevant genes. The samples were rapidly frozen in liquid nitrogen and stored in a −80 °C freezer.

### 2.3. Measurements and Methods

#### 2.3.1. Determination of Physiological Indexes

Superoxide dismutase (SOD) activity was measured following the protocol established by Dhindsa et al. [17]. Peroxidase (POD) activity was assessed based on the method described by Kar et al. [18]. Catalase (CAT) activity was quantified using the UV absorption technique as outlined by the methodology in [19]. Malondialdehyde (MDA) content was determined according to the procedure outlined by Li et al. [20]. Soluble protein content was quantified using the Thomas Brilliant Blue G-250 method [21] (pp. 125–130). Four biological replicates were conducted for each treatment.

#### 2.3.2. Determination of Endogenous Hormones

A total of 50 mg of the sample, which had been finely ground and crushed in liquid nitrogen, was accurately weighed for further analysis. The extraction and quantification of endogenous indole-3-acetic acid (IAA), abscisic acid (ABA), and gibberellins (GAs) were carried out according to the operational guidelines provided by Wuhan Metwell Biotechnology Co., Ltd., China. Data acquisition was performed using ultra-performance liquid chromatography (UPLC) coupled with tandem mass spectrometry (MS) (Shim–pack UFLC-SHIMADZU CBM30A system, Kyoto, Japan; Applied Biosystems, Waltham, MA, USA). Data analysis was conducted by Wuhan Metwell Biotechnology Co., Ltd., Wuhan, China (http://www.metware.cn/) (accessed on 17 November 2021). Three biological replicates were performed for each treatment.

#### 2.3.3. RNA-Seq Analysis

We extracted total RNA from young spikelet tissues using Trizol reagent (Invitrogen, USA), and RNA-seq library construction and sequencing were performed by UW Genetics (Wuhan, China). The differential genes were screened with a Q-value of ≤0.05 and a fold-change (|Log2 ratio|) of >1.5. The FPKM value was used to analyze the expression levels of the differential genes, and Gene Ontology (GO) enrichment analysis was used to analyze the biological processes (agriGO v2.0) in which the differential genes were involved (agriGO v2.0: a GO analysis toolkit for the agricultural community, 2017 update). We performed enrichment analysis using the phyper function in the R software (R 4.2.1) to calculate the *p*-value and then applied FDR correction to the *p*-value, considering a function with a Q-value of ≤0.05 to be significantly enriched.

#### 2.3.4. Quantitative Real-Time PCR

The total RNA was extracted using the E.Z.N.A. Plant RNA Kit (OMEGA, Shanghai, China). The cDNA was synthesized using the Rever Tra AceTM qPCR RT Master Mix with gDNA Remover kit (TOYOBO, Shanghai, China). The qPCR quantitative analysis was performed using a SYBR Green Realtime PCR Master Mix kit (TOYOBO, Shanghai, China). The reaction mix (10 µL) consisted of 5 µL of SYBR Premix Ex Taq, 1 µL of cDNA, 3 µL of ddH_2_O, and 0.5 µL each of the upstream and downstream primers at 10 µmol/µL. The reaction procedure was as follows: 95 °C for 30 s, 95 °C for 5 s, and 60 °C for 15 s. Cycle was repeated 39 times. The relative expression of the genes was calculated using the 2^−ΔΔCt^ method. Three biological replicates were performed for each treatment. The actin gene was used as the internal reference control. All the primers and qRT-PCR sequences used in our study are shown in Supplementary Table S1.

### 2.4. Data Processing and Analysis

We used Microsoft Excel 2020 for data analysis and used Origin 2024 and GraphPad Prism 9.5.1 to draw graphs. We used Duncan’s New Complex Polar Deviation method in the DPS 7.05 statistical software for significance of difference analysis.

## 3. Results

### 3.1. Seed Germination, Antioxidant Enzyme Activity, and Oxidative Damage

**Figure 1:**
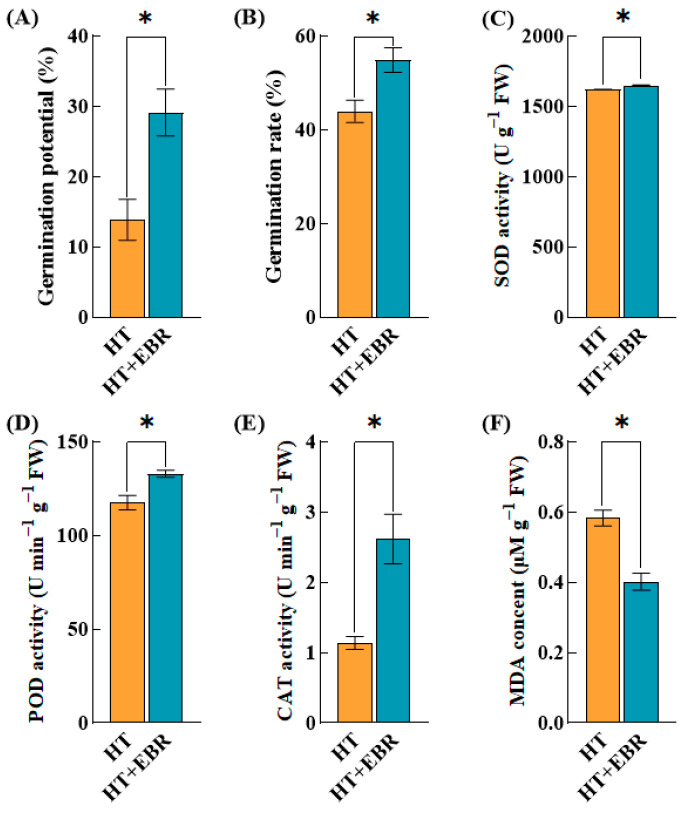
Effect of 0.05 µmol/L EBR dipping on seed germination and antioxidant enzyme system of rice seeds under heat stress. The value corresponding to the histogram on the y-axis is the mean value. A significant difference in the parameters is indicated by the difference in the average value of each letter (±s < 0.05). (**A**) Germination potential; (**B**) germination rate; (**C**) superoxide dismutase (SOD) activity; (**D**) peroxidase (POD) activity; (**E**) catalase (CAT) activity; (**F**) malondialdehyde (MDA) content. The parameter * indicates significant statistical difference, *: *p* < 0.05.

Heat stress adversely affects seed germination through several mechanisms, including the disruption of membrane integrity, alteration of enzymatic activity, induction of oxidative stress, and perturbation of hormonal balance. Collectively, these factors contribute to a reduction in seed vitality and a diminished germination rate. Seed priming with 0.05 µmol/L EBR has been shown to significantly improve the tolerance of rice seeds to heat stress. Specifically, soaking seeds in a 0.05 µmol/L EBR solution at 38 °C led to a notable enhancement in both germination potential and rate, with increases of 110.48% and 24.87%, respectively, relative to the control group (Figure 1A,B).

During seed germination, especially at the stage when the embryo begins to grow, increased cellular metabolic activities may produce large numbers of reactive oxygen species (ROS), such as superoxide (O_2_^•−^), hydroperoxide (H₂O₂), and hydroxyl radicals ^•^OH). Antioxidant enzymes can effectively scavenge these harmful reactive oxygen species, reducing oxidative damage and protecting cellular structure and function. After seed priming with 0.05 µmol/L EBR seeds, the SOD, POD, and CAT activities in rice seeds under heat stress were significantly increased by 1.73%, 13.06%, and 129.81%, respectively (Figure 1C–E).

The MDA content can reflect the degree of oxidative stress experienced by seeds during germination. When seeds experience excessive oxidative stress, MDA accumulation increases. This may damage seed cell membranes and other cellular components, affecting the seed’s ability to germinate. After seed priming with 0.05 µmol/L of EBR, the MDA content of the rice seeds under heat stress was significantly reduced by 31.05% compared with the control treatment (Figure 1F).

### 3.2. Osmotic Regulation Capacity

**Figure 2:**
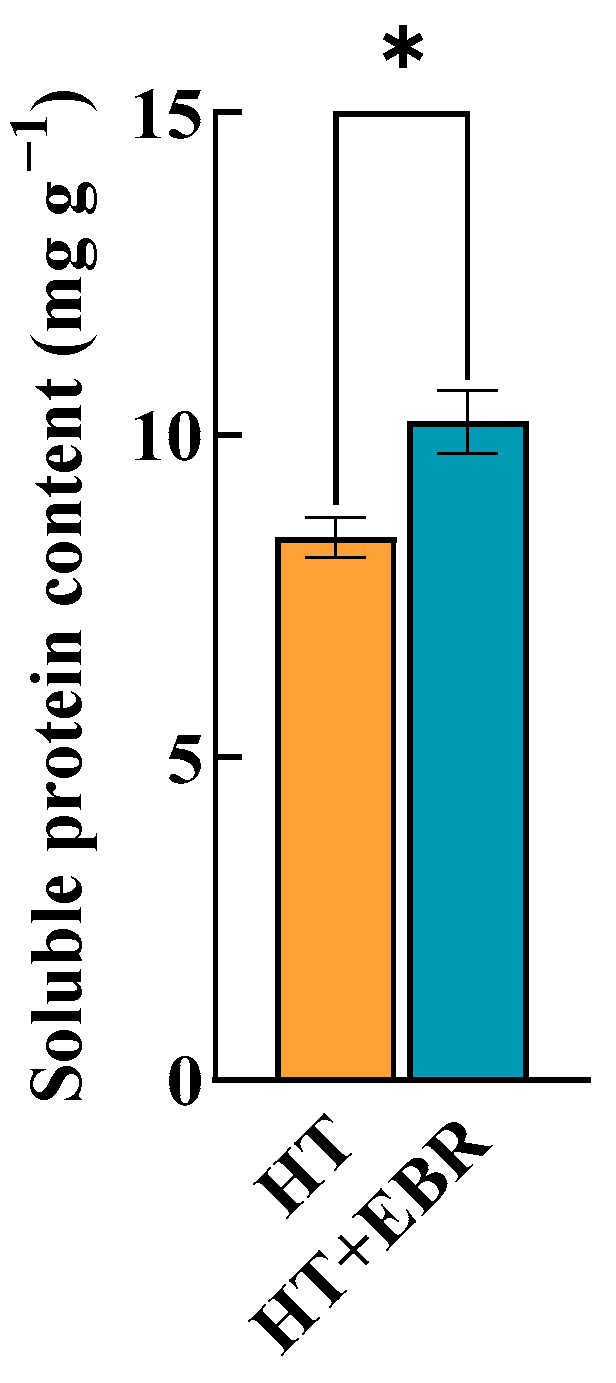
Effect of 0.05 µmol/L EBR dipping on soluble protein content of rice seeds under heat stress. The value corresponding to the histogram on the y-axis is the mean value. A significant difference in the parameters is indicated by the difference in the average value of each letter (±s < 0.05). The parameter * indicates significant statistical difference, *: *p* < 0.05.

Soluble protein changes can effectively reflect the osmoregulatory capacity of rice seeds, with these proteins acting as osmoprotective substances. By regulating the osmotic pressure inside the cell, they help plant cells maintain water balance and prevent cell dehydration. After seed priming with 0.05 µmol/L of EBR, the soluble protein content of rice seeds under heat stress significantly increased by 21.38% compared with the control treatment (Figure 2).

### 3.3. Content of Endogenous Hormones in Seeds

**Figure 3:**
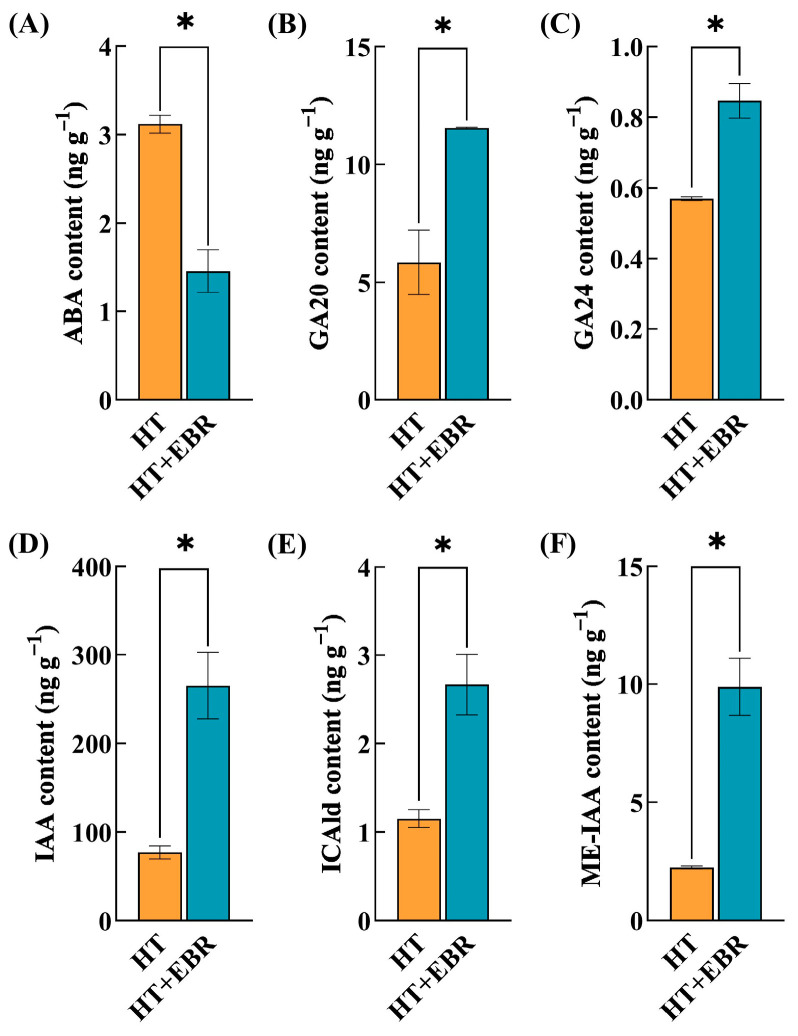
Effect of 0.05 µmol/L EBR dipping on endogenous hormone content of rice seeds under heat stress. The value corresponding to the histogram on the y-axis is the mean value. A significant difference in the parameters is indicated by the difference in the average value of each letter (±s < 0.05). (**A**) ABA content; (**B**) GA20 content; (**C**) GA24 content; (**D**) IAA content; (**E**) ICAld content; (**F**) ME-IAA content. The parameter * indicates significant statistical difference, *: *p* < 0.05.

Plant hormones play a key role in seed germination, regulating the germination process. Meanwhile, the balance between different hormones is crucial for seed germination. When plants recognize adversity stress, plant hormones can participate in secondary metabolic processes by modulating their levels, activating the expression of related genes to resist abiotic stress. After seed priming with 0.05 µmol/L of EBR, the ABA content of rice seeds under heat stress was significantly reduced by 53.21% compared with the control. Both GA20 and GA24 contents were significantly higher than those of the control, at 97.55% and 48.54%, respectively. The ICAld and IAA contents were significantly higher than those of the control by 2.31 and 3.44 times, respectively (Figure 3).

### 3.4. Quality Filtering of Transcriptomic Data

To investigate the mechanism of EBR in alleviating heat stress in rice seeds, we constructed cDNA libraries of seed embryos from the EBR seed priming treatment (HT+EBR) and heat stress treatment (HT) under heat stress and analyzed the transcriptome sequencing. The results showed that the Q20 and Q30 values for both treatments were above 97% and 89%, respectively, indicating high sequencing quality (Supplementary Table S2). After the reads were mapped to the rice reference genome, nearly 90% of the reads could be aligned to the rice reference genome. The sequencing quality for all treatments met the requirements for subsequent transcriptomic analysis.

### 3.5. Gene Expression and Analysis of Differential Genes

**Figure 4:**
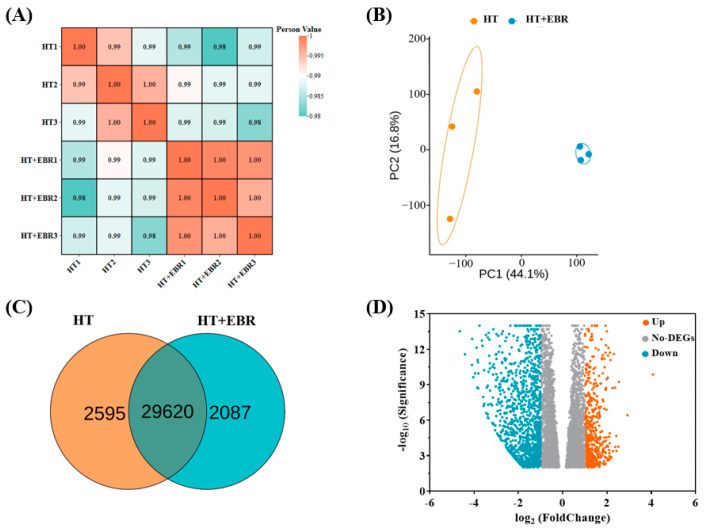
Gene expression analysis of 0.05 µmol/L EBR of rice seeds under heat stress. (**A**) Heat map of correlation between samples; (**B**) PCA analysis of gene expression between groups; (**C**) Venn diagram of gene expression between HT and HT+EBR groups, illustrating the number of genes that are shared and unique among HT and HT+EBR; (**D**) differentially expressed gene volcano map—each point represents a gene. The upregulated genes are denoted by orange dots, the downregulated genes by blue dots, and the genes with no significant difference by gray dots. A gene is considered significantly different under the corrected Q-value if it meets the DEG criteria.

Transcriptome samples contained a large amount of gene expression information. To understand the differences in overall gene expression across groups and the variation within each group, we performed principal component analysis (PCA) to reduce the gene expression data of each sample to a few uncorrelated principal components and compared them between samples. The principal component analysis showed that the samples from the two treatment groups formed two distinct clusters under the two principal components. The first component (PC1) accounted for the largest variability between the two treatments (44.1%), while the second principal component explained 16.8% of the variance (Figure 4B). We found that the three samples from the HT+EBR treatment projected less dispersion on both principal components. By contrast, the three samples from the HT treatment had a greater degree of projection dispersion on the second principal component. However, the correlation coefficients between the three biological replicates of all transcripts from both treatments were above 0.98 (Figure 4A). This indicates that the sample clusters within both treatment groups are highly similar, and the gene expression data are highly reproducible.

A total of 32,215 and 31,707 genes were identified in the HT and HT+EBR treatments, respectively, of which 2595 genes were expressed in the HT treatment, and 2087 genes were expressed in the HT+EBR treatment (Figure 4C). Genes whose differential expression changes by two-fold or more compared with a control under heat stress are usually called differential genes (DEGs). In the differential gene volcano plots, 2903 genes were upregulated, and 3820 genes were downregulated by the EBR seed priming treatment under heat stress (Figure 4D).

### 3.6. GO Pathway Analysis

**Figure 5:**
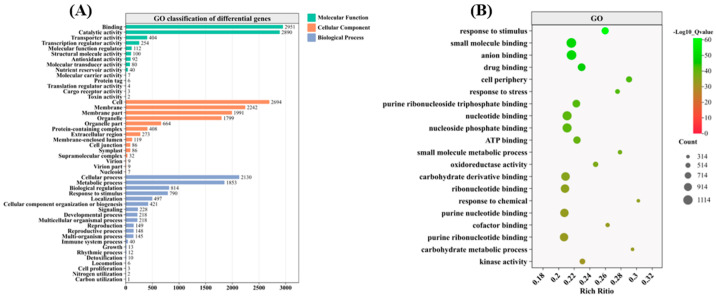
GO term comparison of rice seeds treated with 0.05 µmol/L EBR under heat stress. (**A**) GO classification of differentially expressed genes. (**B**) Bubble plot depicting the GO enrichment of differentially expressed genes.

To further analyze the functional categories of DEGs in the EBR seed-primed treatments under heat stress, we performed functional annotation and GO enrichment analyses of all DEGs. We annotated all DEGs to 48 functional groups within three functional categories: biological processes, cellular components, and molecular functions (Figure 5A). In the biological process category, 20 functional groups were identified, with most DEGs annotated to cellular processes (27.67%), metabolic processes (24.07%), biological regulations (10.57%), and responses to stimulus (10.26%). In the cellular component category, 14 functional groups were identified, with over 80% of DEGs annotated to cells (25.86%), membranes (21.52%), membrane parts (19.11%), and organelles (17.27%). In the molecular function category, 14 functional groups were identified, with most DEGs annotated to binding (42.49%) and catalytic activity (41.61%) (Figure 5B). Based on the GO annotation results, the DEGs were further categorized and enriched, and the 20 most significantly enriched metabolic pathways were selected. Notably, pathways such as response to chemicals, small-molecule binding, anion binding, drug binding, and cell periphery were significantly enriched.

### 3.7. KEGG Pathway Analysis

**Figure 6:**
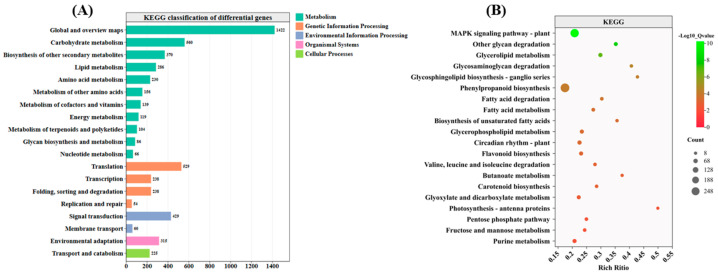
KEGG term comparison of rice seeds treated with 0.05 µmol/L EBR under heat stress. (**A**) KEGG classification of differentially expressed genes. (**B**) Bubble plot showing the KEGG enrichment of differentially expressed genes.

KEGG pathway analysis was conducted to identify the biological pathways enriched by the DEGs. The results revealed that all DEGs were enriched in 19 secondary pathways (Figure 6A). Carbohydrate metabolism, translation, signal transduction, transport, and catabolism had the highest number of DEGs on four branches in metabolism, genetic information processing, environmental information processing, and cellular processes, respectively. The top 20 pathways were analyzed via KEGG enrichment and found to be enriched. Among them, the MAPK signaling pathway—plant, other glycan degradation, glycerolipid metabolism, and glycerolipid metabolism—was significantly enriched (Figure 6B). Seed germination and adaptation to adversity stress were both regulated by plant hormones. Further analysis revealed that DEGs were enriched in the tryptophan metabolism, diterpenoid biosynthesis, carotenoid biosynthesis, and plant hormone signal transduction pathways, with significant enrichment in the synthesis and signaling pathways of IAA, GA, and ABA.

### 3.8. Differential Genes Enriched in Plant Hormone Pathways

**Figure 7:**
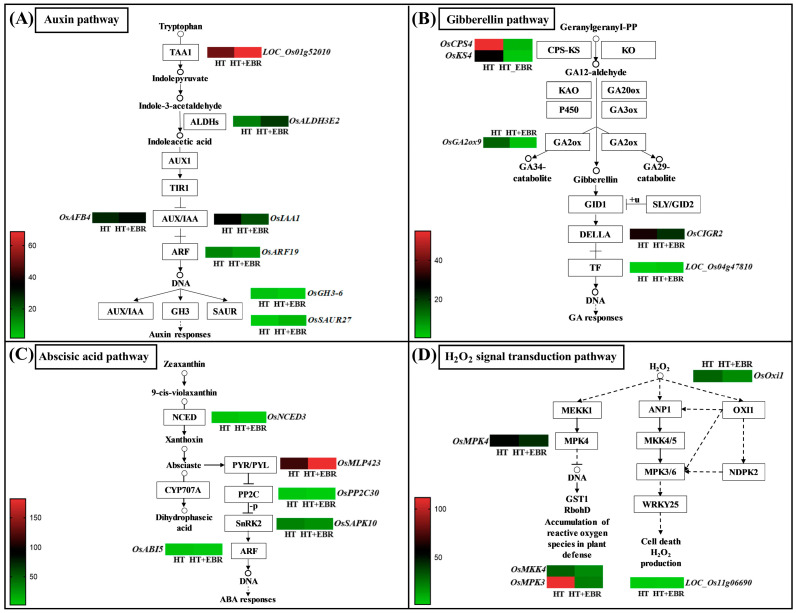
Expression profiles of DEGs in pathways related to IAA, GA, and ABA hormones and reactive oxygen species accumulation. (**A**) Expression profiles of DEGs involved in the IAA pathway under HT and HT+EBR treatments. (**B**) Expression profiles of DEGs related to the GA pathway under HT and HT+EBR treatments. (**C**) Expression profiles of DEGs associated with the ABA pathway under HT and HT+EBR treatments. (**D**) Expression profiles of DEGs connected to the H_2_O_2_ signaling pathway in the plant MAPK signaling pathway under HT and HT+EBR treatments. Sample names are displayed at the bottom or top of the color block. Gene symbols are shown on the left or right side of the color block. Changes in expression levels are indicated by color changes: green indicates a decrease in expression level, and red indicates an increase in expression level. All data are means of three biological replicates (*n* = 3). Means with different letters in each treatment represent significant differences at *p* ≤ 0.05.

Through KEGG pathway analysis, we examined the differentially expressed genes (DEGs) enriched in the IAA, GA, and ABA biosynthesis and signaling pathways in this study. In the IAA biosynthesis pathway, we identified tryptophan aminotransferase (TAA1) and indole-3-acetaldehyde dehydrogenase (ALDH) as two key enzymes involved in IAA synthesis (Figure 7A). The EBR treatment significantly upregulated the expression of the *LOC_Os01g52010* and *OsALDH3E2* genes, which regulate these enzymes, thereby promoting IAA synthesis. In the IAA signaling pathway, significant upregulation of the *OsAFB4* gene, which regulates transport inhibitor response 1 (TIR1), was observed upstream of the signaling cascade. Midstream in the signaling pathway, EBR significantly downregulated *OsIAA1* and *OsARF19*, genes from the Aux/IAA family and auxin response factors, respectively. At the terminal of the signaling pathway, EBR significantly downregulated the expression levels of the IAA inactivation-related *OsGH3-6* genes, inhibited IAA inactivation, and further promoted IAA levels in the rice seeds. Meanwhile, EBR significantly upregulated the expression level of *OsSAUR27* in the SAUR family at the end of IAA signal transduction, which enhanced IAA signal output.

The biosynthesis of GA can be divided into three steps. The first step involves forming the precursor molecule for GA synthesis, geranylgeranyl pyrophosphate (GGPP). The second step is synthesizing GA12-aldehyde (Gibberellin A12 aldehyde). Finally, GA12-aldehyde is converted into other GAs. In the GA synthesis pathway, we found that EBR significantly downregulated the expression levels of the *OsCPS*4 gene, which regulates homotrimeric corbasic pyrophosphate synthase, and the *OsKS4* gene, which controls the expression of 9β-hexahydro-7,15-diene synthase. This downregulation affected the first step of GA biosynthesis, limiting the synthesis of GA12-aldehyde and consequently restricting GA biosynthesis (Figure 7B). Furthermore, we observed that EBR significantly downregulated the expression of *OsGA2ox9*, a gene encoding the gibberellin 2β-dioxygenase responsible for GA inactivation, thus inhibiting GA deactivation. In the signal transduction pathway of GA, EBR significantly upregulated the expression level of phytochrome interaction factor 4 (PIF4), and the GA output signal was enhanced.

9-cis-epoxycarotenoid dioxygenase (NCED) is considered the key rate-limiting enzyme in the ABA synthesis process. Our results revealed that EBR significantly downregulated *OsNCED3* expression levels and slowed down the rate of ABA synthesis (Figure 7C). In the signal transduction pathway of ABA, we observed that EBR induced the expression of differential genes upstream, midstream, and downstream; most of these differential genes were significantly downregulated at the expression level. In the upstream region of ABA signaling, EBR significantly upregulated the *OsMLP423* latex protein. In the midstream of ABA signaling, EBR significantly downregulated the genes encoding protein phosphatase 2C (*OsPP2C30*) and stress-activated protein kinase (*OsSAPK10*). In the downstream region of ABA signaling, EBR significantly downregulated the expression of *OsABI5*, a gene from the *bZIP* transcription factor family.

### 3.9. Differential Genes Enriched in Reactive Oxygen Species Signaling Pathways

In the KEGG pathway analysis, we found that DEGs were enriched in the H_2_O_2_ signaling pathway in the plant MAPK signaling pathway (Figure 7D). H_2_O_2_ is a key plant signaling molecule and induces the MAPK cascade in response to adversity stress. External stimuli and high-pressure environments disrupt the balance of reactive oxygen species (ROS) in plants, leading to H_2_O_2_ accumulation, which subsequently triggers H_2_O_2_-mediated signaling pathways. In the three H_2_O_2_-mediated pathways, we observed that EBR significantly downregulated the expression levels of five genes: *MPK4*, *MKK4/5*, *MPK3/6*, *NDPK2*, and *OxI1*.

### 3.10. Validation of DEGs Using qRT-PCR

**Figure 8:**
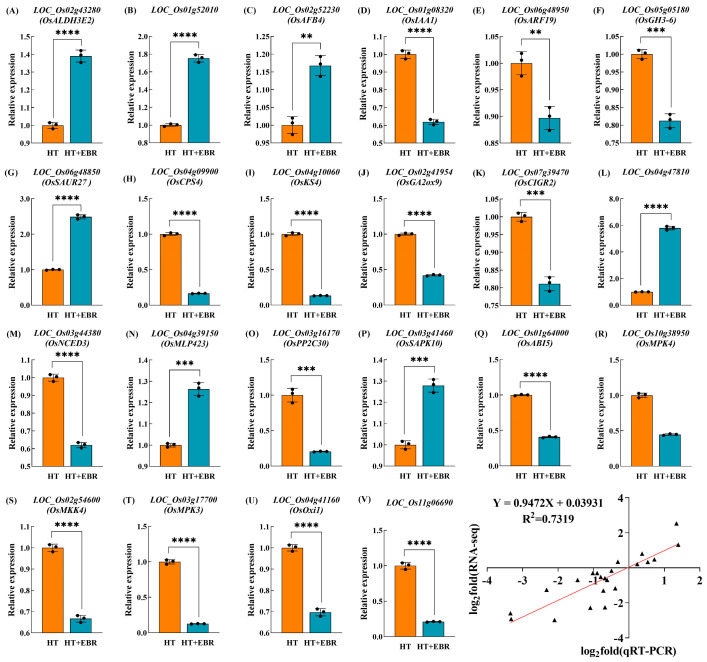
The expression of 22 differentially expressed genes involved in the IAA, GA, ABA, and H_2_O_2_ pathways was randomly validated by qRT-PCR. (**A**−**G**) IAA-responsive genes; (**H**−**L**) GA-responsive genes; (**M**−**Q**) ABA-responsive genes; (**R**−**V**) H_2_O_2_ production genes. The x-axis represents the treatment name, where HT refers to the 38 °C EBR non-soaked treatment and HT+EBR refers to the 38 °C EBR soaked treatment. The y-axis indicates the relative expression of a specific gene compared to the reference gene actin. The final panel displays the results of the RNA-seq/qRT-PCR correlation analysis (**bottom right**). The x-axis shows the log2 fold change value for qRT-PCR, and the y-axis shows the log2 fold change value for RNA-seq. A significant positive correlation was observed between the changes in expression, according to the Pearson correlation (R^2^ = 0.7319; *p* < 0.05). Statistical significance is indicated by *, ** 0.001 < *p* < 0.01, *** 0.0001 < *p* < 0.001, **** *p* < 0.0001.

To verify the accuracy and reproducibility of the RNA-seq results, we selected 22 genes in the phytohormone synthesis, metabolism, and signaling pathways and the H_2_O_2_ signaling pathway for qRT-PCR analysis. The expression levels of 22 genes in the HT+EBR treatment showed significant differences compared with the HT treatment (Figure 8). The expression of 15 genes was significantly downregulated, and 7 genes were significantly upregulated. By comparing the correlation between the RNA-Seq results and qRT-PCR results (2^−ΔΔCt^) of the 22 DEGs, we detected that the RNA-Seq and qRT-PCR results for the 22 DEGs were significantly similar (R^2^ = 0.7319). This demonstrates that the RNA-seq data are reliable and accurate.

## 4. Discussion

### 4.1. Effect of EBR on Physiological Changes in Rice Seeds Under Heat Stress

Seed germination is essentially a process that activates a series of metabolic activities. Under suitable environmental conditions, seeds receive signals to initiate germination. These signaling molecules are transmitted between cells, subsequently triggering specific metabolic pathways associated with germination. Reactive oxygen species (ROS) are key secondary messengers during seed germination. Through their redox properties, ROS transmit signals to the nucleus, promoting the degradation of polysaccharides and the carbonylation of DNA, RNA, fatty acids, and proteins, thereby accelerating the germination process. Following water absorption and seed swelling, increased ROS levels are usually regarded as a signal of germination initiation [22]. However, seed germination has a strict requirement limit for ROS concentrations. Both excessively low and high concentrations can inhibit or delay germination. When ROS accumulation exceeds the tolerance threshold of the cell’s defense mechanisms, oxidative stress occurs, which may lead to seed dormancy or even death. Notably, ROS are natural by-products of plant cell metabolic activities and are generated in separate organelles such as chloroplasts, mitochondria, cytoplasm, and peroxisomes [23]. Various abiotic stress conditions, such as heat stress, can indirectly lead to the accumulation of excessive ROS by disrupting normal cellular metabolic activities, thereby triggering cellular oxidative stress. Therefore, it is important to control the level of ROS in seed cells under heat stress within the “oxidative window” of seed germination to alleviate oxidative stress in seed cells and to promote germination under increasing global temperatures.

According to our experiment, when the rice seeds were initiated by 0.05 µmol/L of EBR, their MDA content under heat stress was significantly lower by 31.05% compared with the control. This indicates the positive role of EBR in mitigating the lipid peroxidation of cell membranes in rice seeds. This positive effect helped to maintain the segregated state of the cell and membrane integrity, guaranteeing the normal activity and function of various enzymes and organelles in the cell. Moreover, SOD, POD, and CAT activities were significantly increased by 1.73%, 13.06%, and 129.81% in the rice seeds under heat stress compared to the control. This suggests that EBR helps regulate ROS levels within the “oxidative window” for seed germination by scavenging excess ROS in the seeds. On the one hand, reducing the ROS content in seeds under heat stress to an optimal level continues to activate the signaling cascade for seed germination and mediates the programmed cell death of the aleurone layer, providing sufficient nutrients for seed germination [24]. On the other hand, alleviating the oxidative stress state in seeds reduces oxidative damage to cellular DNA. Activating DNA repair mechanisms is one of the most valuable and critical processes triggered during seed germination [25]. This process is usually activated during the first stage of seed germination, the stage of rapid water absorption and swelling, and is essential for the recurrence of cell cycle activity. However, heat stress disrupts the DNA repair process and cell cycle initiation, leading to nuclear DNA breaks and arresting cell division. This type of DNA damage, resulting from the disruption of the structural or functional integrity of the genome, is referred to as genotoxic stress [26]. In a previous study, the initiation technique effectively promoted DNA repair and enhanced DNA metabolism and transcriptional activity to avoid genotoxic stress in plants [27]. For example, Tribulus terrestris seeds underwent hydroinitiation, significantly upregulating the expression levels of genes involved in DNA damage repair and antioxidant mechanisms and significantly increasing the activity of formamidopyrimidine DNA glycosylase (FPG), which is involved in base excision repair [28]. In addition, heat-stress-induced DNA damage inhibits cell elongation and downregulates the expression of some cell cycle protein genes, resulting in cell cycle arrest [29]. Seed germination relies on cell division and expansion. Brassinosteroids (BRs) induce cell elongation by directly targeting the expression of CESA genes [30]. BRs also regulate the cell cycle and the proliferation of the quiescent center (QC) in the root [31]. The QC is a tissue center located at the junction between the root cap and the meristematic zone. It maintains root growth primarily through asymmetric division, which continuously self-renews and produces daughter cells that can differentiate into various types of root cells. BR formation is a sign of the embryonic root’s initiation. Under stress conditions, BRs promote QC division by inhibiting the negative regulators of QC [32,33]. In repeated repair processes, the accumulation of specific proteins and the activation of related genes are stored as “stress memories” in plants. When plants are re-exposed to the same type of stress, the stored stress memory helps the plant to respond quickly and recover rapidly from the damage caused by this stress [34]. This may explain why the beneficial effects of seed priming during the seed germination phase can persist into later stages of plant growth. In conclusion, we hypothesize that EBR alleviates oxidative stress in rice seeds under heat stress by enhancing the activity of antioxidant enzymes to control intracellular ROS levels and promoting seed germination by stimulating cell division and expansion. We also hypothesize that heat stress memory stored at the seed germination stage improves heat tolerance in later stages.

Using KEGG analysis, we found that EBR induced the differential expression of numerous genes in the MAPK (mitogen-activated protein kinase) signaling pathway in plants. Composed of a series of Ser/Thr protein kinases, the MAPK cascade is a critical pathway for cells to respond to various external signals and trigger appropriate cellular responses. It plays a vital role in the stress response of eukaryotes. Hydrogen peroxide (H_2_O_2_), as a key plant signaling molecule, can trigger the MAPK cascade to cope with heat stress. Under heat stress, the expression levels of MAPKs typically increase significantly. For instance, in *Chrysanthemum morifolium*, heat stress rapidly and significantly upregulates *CmMPK4.1*, *CmMPK6*, and *CmMPK13* in leaves [35]. Similarly, in lettuce, most *LsMAPKs* respond to heat stress, with a particularly significant increase in the expression level of *LsMAPK4* [36]. The KEGG analysis results showed that the EBR treatment significantly reduced the expression levels of genes in several MAPK cascade pathways activated by H_2_O_2_ under heat stress conditions. These genes included *MKK4/5*, *MPK3/6*, *NDPK2*, and *OxI1*, the expression of which is associated with cell death and ROS production. Additionally, EBR significantly downregulated the expression levels of *MPK4* genes, which are closely associated with reactive oxygen species accumulation in plant defense responses. This further reduced H_2_O_2_ production in rice seeds under heat stress. Furthermore, the downregulation of these genes’ expression levels laterally verified the effective alleviation of oxidative stress using EBR by enhancing antioxidant enzyme systems.

Seed germination is a complex, multi-stage process. Water absorption is crucial for activating the physiological activities and metabolism of the seed. Thermal stress can damage the structure and function of cell membranes, including their fluidity, permeability, thickness, and stacking, leading to an imbalance in osmotic pressure both inside and outside the cell and resulting in cellular dehydration [37]. In response to this stress, cells initiate defense mechanisms to produce and accumulate low-molecular-weight organic compounds, known as compatible solutes, to maintain osmotic pressure homeostasis [38]. The higher concentration of soluble protein, a type of compatible solute, in the cell indicates a stronger osmoregulatory capacity [39]. In our study, after priming the seeds with 0.05 µmol/L of EBR, the soluble protein content in rice seeds under heat stress significantly increased by 21.38% compared with the control. This suggests that EBR effectively enhances the osmotic regulation capacity of rice seeds under heat stress, improves the cells’ water retention ability, and thereby promotes seed germination.

### 4.2. Effect of EBR on the Synthesis and Metabolism of Plant Hormones in Rice Seeds Under Heat Stress

Plants regulate genes in hormone synthesis and metabolic pathways to control the levels of endogenous hormones. A KEGG pathway analysis revealed that EBR induced many differentially expressed genes (DEGs) in the synthesis and metabolism pathways of IAA, GA, and ABA under heat stress, including those related to tryptophan metabolism, diterpenoid biosynthesis, and carotenoid biosynthesis. Tryptophan is a precursor in the biosynthesis of auxin. In the tryptophan (TAM) pathway, we found that EBR significantly induced the expression levels of two genes, *TAA1* and *ALDHs*, which promoted IAA biosynthesis. The increased IAA levels activate the plasma membrane H-ATPase (PM H-ATP) proton pump, accelerating the extrusion of protons (H). This decreases the apoplast pH and alters the activity of cell-wall-modifying proteins, enhancing the extensibility of the cell wall and increasing the rate of cell expansion, thereby promoting seed germination [40]. GA is a class of tetracyclic diterpenoid compounds. In the diterpenoid biosynthesis pathway, we found that EBR significantly downregulated *OsCPS4* and *OsKS4* expression levels, limiting GA synthesis. At the same time, EBR also significantly downregulated the expression of *OsGA2ox9*, which promotes GA inactivation. This indicated that EBR enhances the GA content in seeds by regulating both GA synthesis and metabolic pathways rather than through a single pathway. In the ABA biosynthesis pathway, EBR significantly downregulated *OsNCED3*, a key rate-limiting enzyme in ABA biosynthesis, resulting in the suppression of ABA synthesis and decreased ABA content in seeds. The increased GA content and decreased ABA content led to an elevated GA/ABA ratio. In conclusion, EBR alleviated the inhibitory effect of heat stress on rice seed germination by regulating the differential expression of genes in the IAA, GA, and ABA synthesis and metabolic pathways, thus increasing the IAA expression level and GA/ABA ratio in seeds.

### 4.3. Effect of EBR on Regulation of Phytohormone Signaling Pathways in Rice Seeds Under Heat Stress

Plasticity properties exhibited by plants during their intrinsic development are mainly regulated by hormonal signaling pathways. These pathways not only facilitate the harmonious functioning of their internal developmental programs but also translate signals from environmental factors into the plant. A KEGG analysis revealed that EBR induced the expression of DEGs in the IAA, GA, and ABA signaling pathways. Auxin signaling is primarily mediated by transport inhibitor response 1 (*TIR1*), auxin/indole-3-acetic acid (Aux/IAA) proteins, and auxin response factors (ARFs) [41]. Auxin promotes the degradation of Aux/IAA proteins by stabilizing the interaction between *TIR1* and the second structural domain of Aux/IAA, thereby enabling the release of ARF from the Aux/IAA-ARF dimer, ultimately transmitting the IAA signal [42,43]. In this experiment, EBR upregulated the expression of the *OsAFB4* gene, which regulates *TIR1*, and downregulated the expression of *OsIAA1*, which regulates Aux/IAA, and *OsARF19*, which regulates ARF, in response to heat stress. The differential expression of *OsAFB4* and *OsIAA1* indicates an enhancement of IAA signal output. By contrast, the ARF expression level did not increase in response to the enhanced IAA signal. This could be related to the significant decrease in the expression of *OsGH3-6*, a member of the GH3 family, which acts downstream of ARF. The GH3 family encodes a class of enzymes that catalyze the conjugation of IAA with amino acids, inactivating IAA through aminoacylation. This has been validated in vitro through reactions involving Arabidopsis GH3 proteins and various plant auxins [44]. ARFs can positively regulate the expression of GH3 genes [45]. Under heat stress, the EBR-induced downregulation of *OsARF19* expression inhibited IAA inactivation mediated by *OsGH3-6*, thereby further increasing the IAA levels in the seeds. Additionally, we observed that EBR not only induced the differential expression of GH3 family genes but also significantly upregulated the expression of *OsSAUR27*, a member of the SAUR family, among early auxin response genes. This is associated with enhanced IAA signal output. SAUR, as an early auxin response gene, is involved in regulating cell elongation, and SAUR overexpression can promote cell elongation in Arabidopsis [46]. Therefore, EBR promotes the elongated growth of rice seed cells under heat stress by increasing the IAA expression level and rapidly inducing the expression of *OsSAUR27*. GA positively regulates seed germination. In the GA signaling pathway, DELLA proteins act as negative regulators. DELLA proteins can interact with Phytochrome-Interacting Factor 4 (PIF4) and inhibit GA signal output by preventing PIF4 from binding to the promoters of target genes [47]. GA mediates the degradation of DELLA proteins by binding to *GID1*, thereby allowing GA signal output [48]. Under heat stress, EBR inhibited the expression of DELLA and increased the expression level of PIF4, which enhanced the output of GA signals. The enhanced GA signal output under heat stress promoted rice seed germination.

ABA inhibits seed germination. The core components of ABA signal transduction include the ABA receptors PYR/PYL/RCAR, type 2C protein phosphatases (PP2C), and SNF1-related protein kinase 2 (SnRK2). Among these, PP2C acts as a negative regulator in the ABA signaling pathway. In the absence of ABA, PYL proteins release PP2C, which then inhibits the activity of downstream ABA signaling proteins through protein dephosphorylation, thereby suppressing the function of the downstream ABA signaling network [49]. According to our experiments, EBR significantly upregulated the expression of the *OsMLP423* ABA receptor and downregulated the expression of *OsPYL9* under heat stress. *OsPYL9* positively regulates ABA response. Downstream of PYR/PYL action, the expression of *OsPP2C30*, a member of the PP2C family, was significantly upregulated by EBR. The downregulation of *OsPP2C30* expression suggests enhanced ABA signal output. However, EBR also significantly downregulated the expression of key genes involved in ABA-mediated seed dormancy signaling, including *OsSAPK10* and *OsABI5*. The expression of these genes obscures the intensity of ABA signal output. However, given a combination of crosstalk between the expression of a number of genes in other hormonal pathways and hormonal signaling, we hypothesized that EBR restricts the output of ABA signaling under heat stress, thereby promoting seed germination. It thus released ABA signaling-mediated seed dormancy by inhibiting the expression of *OsPYL9*, *OsSAPK10*, and *OsABI5*. The upregulation of *OsMLP423* expression induced by EBR is more related to its involvement in mitigating oxidative stress caused by heat stress. *OsMLP423* expression can physiologically reduce membrane damage and reactive oxygen species accumulation. *OsMLP423* overexpression plants have shown significantly higher SOD, POD, and CAT enzyme activities and lower O_2_^−^, H_2_O_2_, and MDA contents [50]. Downregulated PP2C expression may be related to the enhancement of SAUR expression in the IAA signal transduction pathway, an inhibitor of PP2C protein kinase. In conclusion, EBR promotes rice seed germination and enhances the seed’s antioxidant enzyme system under heat stress by regulating the differential expression of five genes: *OsPYL9*, *OsMLP423*, *OsPP2C30*, *OsSAPK10*, and *OsABI5*.

### 4.4. Effect of EBR on Redundant Interactions Between Plant Hormones in Rice Seeds Under Heat Stress

**Figure 9:**
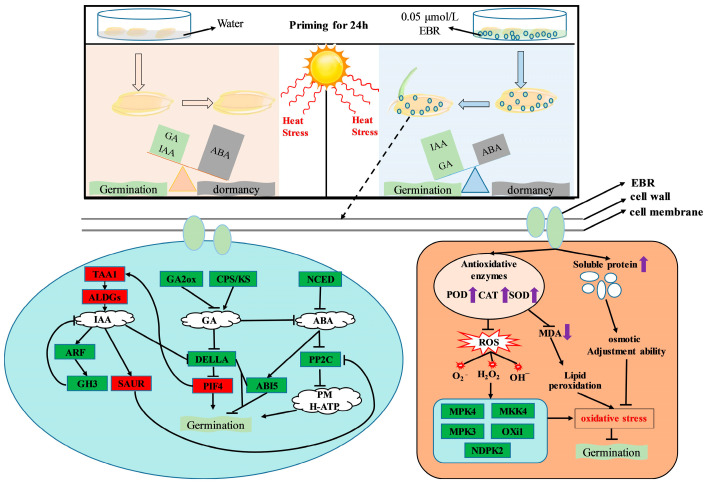
A 0.05 µmol/L EBR treatment used as a seed priming agent enhanced the vigor of rice seeds under heat stress.

Genes extensively interact in the plant hormone signaling pathway. These genes act up and down and are hierarchically linked, constituting the phytohormone signaling network. Previous studies have shown that the auxin-mediated acidic growth mechanism is regulated by SAUR and is associated with the negative regulator PP2C in ABA signaling. In this growth mechanism, SAUR inhibits PP2C.D activity, preventing it from dephosphorylating the PM H-ATPase (such as AHA), thereby restoring proton pump activity, acidifying the cell wall, and ultimately promoting cell elongation [51]. Interestingly, EBR reduced the expression levels of *OsSAPK10* and *OsABI5* genes in the ABA signaling pathway, which are closely related to seed dormancy, restricting ABA signaling. Meanwhile, the downregulation of *OsPP2C30* gene expression indicated the partial activation of the ABA signaling pathway. The regulation of ABA signaling induced by EBR promotes the awakening of seeds from dormancy while allowing a certain degree of ABA signaling to be released, triggering the seed defense mechanism and thus enhancing seed tolerance to heat stress. This demonstrates that EBR fine-tunes the ABA signaling pathway under heat stress conditions, balancing the relief of seed dormancy and the activation of defense mechanisms. The PIF4 factor can upregulate the expression of the key auxin biosynthesis gene *TAA1*, thereby promoting auxin biosynthesis [52]. Furthermore, increased auxin levels exert an inhibitory effect on the negative regulator DELLA in the GA signaling pathway, further enhancing GA signal transduction [53]. Correspondingly, the expression of PIF4 is positively regulated by GA signaling, forming a positive feedback loop that further drives auxin synthesis. Under heat stress, EBR effectively promotes seed germination through this inter-hormonal positive feedback mechanism. Notably, the RGL2 factor within the DELLA protein family plays a crucial role in seed dormancy, which is dependent on the activity of the ABI5 factor. RGL2 activates the promoter region of the ABI5 gene through cooperative interaction with three nuclear factors, NF-YC3, NF-YC4, and NF-YC9, thereby mediating seed dormancy [54]. These findings reveal the pivotal signaling hub role of DELLA proteins within the EBR-regulated hormone signaling network. According to our results, EBR can regulate the antioxidant system, as well as the synthesis, transduction, and interaction networks of plant hormone signals, enabling adaptation to changes in the external environment under heat stress. At a concentration of 0.05 µmol/L, EBR enhanced the vigor of rice seeds under heat stress as a seed priming agent (Figure 9). In the future, we will investigate the molecular regulatory mechanism of EBR in the heat tolerance of rice seeds.

## 5. Conclusions

As a seed initiator, EBR has broad application prospects and can improve agricultural production efficiency and crop quality by promoting seed germination, enhancing crop stress resistance, improving root growth, increasing yield and quality, and promoting other pathways. Our experiments showed that 0.05 µmol/L of EBR, as a seed priming agent under heat stress, precisely regulates seed germination through the redundant effects of key plant hormone genes and the MAPK signaling cascade. This regulation is achieved by coordinating changes in antioxidant enzyme activity, MDA content, soluble protein content, and plant hormone levels. Our study provides new insights into the role of EBR in modulating plant hormone signaling networks and its function in plant stress responses, offering a feasible solution for the application of seed priming techniques. Future research on EBR will focus more on the application effects and mechanisms of different crops, exploring their optimal use under different soil types and climatic conditions. Meanwhile, with the increasing demand for green agricultural development, EBR is expected to reduce dependence on chemical fertilizers and pesticides, promoting sustainable agricultural development. In addition, EBR can improve seed storage tolerance and sowing adaptability, promote seed storage and transportation, and provide more flexible solutions for agricultural production.

## Data Availability

All of the data is contained within the article and the Supplementary Materials.

## Supplementary Materials

The following supporting information can be downloaded at https://www.mdpi.com/article/10.3390/antiox14020242/s1. Table S1: Primers for qRT-PCR expression analysis. Table S2: Output and quality of sample sequencing data.
