# Supplementary material for: Seed Priming with 2,4-Epibrassionolide Enhances Seed Germination and Heat Tolerance in Rice by Regulating the Antioxidant System and Plant Hormone Signaling Pathways
Source: Antioxidants (Basel). 2025 Feb 19;14(2):242. doi: 10.3390/antiox14020242 (PMC11851696; doi:10.3390/antiox14020242)
Supplement: Supplementary file 1 [file antioxidants-14-00242-s001.zip › antioxidants-3439979-supplementary.pdf]

Table S1. Primers for qRT-PCR expression analysis

Gene Name	cDNA Accession No.	Forward Primer	Reverse Primer	Description
LOC_Os02g43280(OsALDH3E2)	AK120274	TACGCCTTCACCAACAAC	CTGTCCGAATCCGCTATG	*OsALDH3E2* encodes an aldehyde dehydrogenase enzyme, which plays a crucial role in detoxifying reactive aldehydes produced during oxidative stress. It is involved in the plant's response to environmental stress by neutralizing harmful aldehyde compounds.
LOC_Os01g52010	AK064938	GCCTCACCTGACGCACATCC	CGCCACCTTCTCGTCCCTTAT	The specific function of this gene is not well characterized, but it is likely involved in plant growth, development, or stress response pathways.
LOC_Os02g52230(OsAFB4)	AK073912	TCATCGCCAAGTCATTCC	CCTCCTCGTCAATATAGTCTT	*OsAFB4* encodes a member of the Auxin F-Box protein family. It plays a significant role in auxin signaling, a key plant hormone that regulates growth processes such as cell division, elongation, and root development.
LOC_Os01g08320(OsIAA1)	AK109373	GCCTACTCCGGCTACGACCA	CGTACTCCGTCCCGTTCACC	*OsIAA1* encodes an Aux/IAA protein, which is a critical component in the auxin signaling pathway. This protein regulates various aspects of plant growth, including root development and response to environmental stimuli.
LOC_Os06g48950(OsARF19)	AB071296	CCGACAGCCAACTAACATA	CATAGACTGCCTTCTGATACT	*OsARF19* encodes an Auxin Response Factor (ARF) that mediates the auxin signal transduction pathway. It is involved in regulating gene expression in response to auxin, influencing processes such as root formation and stress responses.
LOC_Os05g05180(OsGH3-6)	AK106538	CACCTCCTACGACGACTA	CGAATCCAAGTAGGGTATCA	*OsGH3-6* encodes a protein from the GH3 family, which is involved in the conjugation of auxins and other phytohormones. It helps regulate plant hormone levels and contributes to growth regulation and stress adaptation.
LOC_Os06g48850(OsSAUR27)	AP003726	GGTGTAGGCAGGGCGAGCAA	GATCACGAACCGCCGCATCT	*OsSAUR27* encodes a protein that is part of the SAUR (Small Auxin Up RNA) family. This gene is involved in mediating auxin-induced cell elongation and plays a role in the plant's response to growth signals and environmental stress.
LOC_Os04g09900(OsCPS4)	AB066270	CGCTCATTCCTCCAAGAC	ATCGCCACTACCTCCATAT	*OsCPS4* encodes a carotenoid pathway enzyme, specifically a diterpene cyclase involved in the biosynthesis of gibberellins, which are plant hormones that regulate growth and development.
LOC_Os04g10060(OsKS4)	AB126934	CAGCCAGCAAGGATATTCT	AATGTGAGGTTGAAGCCTAT	*OsKS4* encodes a gene that is involved in the biosynthesis of gibberellins, contributing to plant growth regulation, seed germination, and flowering time.
LOC_Os02g41954(OsGA2ox9)	AK108598	CGGCGACCTCACCTCCCTCA	TGGTCCTGGCAGAGCACGGT	*OsGA2ox9* encodes an enzyme involved in the metabolism of gibberellins by inactivating them. It plays a role in regulating plant growth, particularly in the control of stem elongation and seed development.
LOC_Os07g39470(OsCIGR2)	AK073470	CCACATTCCAGACGAGTC	GAAGATGGCGGTGTAGTAG	*OsCIGR2* encodes a protein that is involved in maintaining cell wall integrity and regulating cellular responses to stress. It may play a role in plant defense mechanisms.
LOC_Os04g47810	AK109094	ACACAGCCTTCTCATCGT	CTCTCGTGTTGTCAAGCA	The function of this gene is not fully characterized, but it may be involved in various biological processes such as growth regulation or stress response.
LOC_Os03g44380(OsNCED3)	AY838899	TCAAGAAGCCGTACCTCA	CGTAGTTCTCGGTGATGG	*OsNCED3* encodes a key enzyme in the biosynthesis of abscisic acid (ABA), a plant hormone critical for stress responses, particularly under drought and other environmental stress conditions.
LOC_Os04g39150(OsMLP423)	AK059231	CGAGCAGTACAAGAGCAT	CGACACCACCTTCTTCTC	*OsMLP423* encodes a member of the maize lipid transfer protein (MLP) family, which may be involved in stress responses by mediating lipid transfer and influencing cell membrane stability.
LOC_Os03g16170(OsPP2C30)	AK069274	CCCGCGTCTTTGGCATGCTC	GCTCACCACGTCCCACAGGC	*OsPP2C30* encodes a protein phosphatase 2C, which is involved in ABA signaling. It plays a role in the regulation of plant stress responses, particularly under drought conditions.
LOC_Os03g41460(OsSAPK10)	AB125311	TGTCCACATATCTCCAGAGT	TGCTATCATCCATAAGGTCAG	*OsSAPK10* encodes a stress-activated protein kinase that is involved in signaling pathways related to abiotic stress responses. It helps regulate cellular processes under stress conditions like drought or heat.
LOC_Os01g64000(OsABI5)	EF199630	GGATGATGATTGAGAATGGA	TGCTTGAGATAGTTCAGTTC	*OsABI5* encodes a transcription factor that plays a key role in the ABA signaling pathway, particularly in the regulation of seed dormancy, germination, and stress responses.
LOC_Os10g38950(OsMPK4)	EF174189	GAACTTCTTCGAGGTCTCC	CTTCTTGATGGCAACTTCC	*OsMPK4* encodes a mitogen-activated protein kinase (MAPK), which is involved in transducing environmental signals. It plays a critical role in the regulation of stress responses, as well as in plant development.
LOC_Os02g54600(OsMKK4)	AK120525	GGACCATCGCCTACATGA	CCTGCTTGCCGAGATTCT	*OsMKK4* encodes a MAPK kinase that activates downstream MAPKs. It is involved in regulating plant stress responses, including those related to pathogen attack and environmental stress.
LOC_Os03g17700(OsMPK3)	AJ486975	TCACATCATCCGCTCCAA	ATCTTGAGGTCGCAGTTG	*OsMPK3* is a MAPK gene, involved in signaling pathways that regulate stress responses, including heat and pathogen resistance.
LOC_Os04g41160(OsOxi1)	AK067266	CCAGACCGAGAAGATGTT	GGCATGGAATCAGCAATC	*OsOxi1* encodes a protein involved in oxidative stress response. It functions as a redox-regulated protein that contributes to the plant's ability to tolerate stress by mitigating oxidative damage.
LOC_Os11g06690	AC120527	ATTATCAGAAGTGCCCTAGC	GACCAGAGTTGAACAGAGAA	The specific function of this gene is not well characterized, but it is likely involved in plant growth, development, or stress response pathways, particularly in response to biotic or abiotic stress.

Table S2. Output and quality of sample sequencing data

Sample	Total Raw Reads (Mil.)	Total Clean Reads (Mil.)	Total Clean Bases (Gb)	Clean Reads Q20(%)	Clean Reads Q30(%)	Clean Reads Ratio (%)
HT1	45.29	45.14	6.77	97.19	89.62	99.67
HT2	45.40	45.24	6.79	97.19	89.61	99.65
HT3	43.92	43.89	6.58	97.2	89.72	99.93
HT+EBR1	43.83	43.8	6.57	97.33	90	99.93
HT+EBR2	43.87	43.84	6.58	97.35	90.11	99.92
HT+EBR3	43.87	43.83	6.57	97.39	90.22	99.92
